# Conventional and new-breeding technologies for improving disease resistance in lentil (*Lens culinaris* Medik)

**DOI:** 10.3389/fpls.2022.1001682

**Published:** 2023-01-20

**Authors:** Anirban Roy, Parmeshwar K. Sahu, Camellia Das, Somnath Bhattacharyya, Aamir Raina, Suvendu Mondal

**Affiliations:** ^1^ Department of Genetics and Plant Breeding, Bidhan Chandra Krishi Viswavidyalaya, Mohanpur West Bengal, India; ^2^ Department of Genetics and Plant Breeding, Ramakrishna Mission Vivekananda Educational & Research Institute (RKMVERI), Ramkrishna Mission Ashrama, Kolkata, India; ^3^ Department of Genetics and Plant Breeding, College of Agriculture, Indira Gandhi Krishi Viswavidyalaya, Raipur, Chhattisgarh, India; ^4^ Mutation Breeding Laboratory, Department of Botany, Aligarh Muslim University, Aligarh, Uttar Pradesh, India; ^5^ Botany Section, Women’s College, Aligarh Muslim University, Aligarh, Uttar Pradesh, India; ^6^ Nuclear Agriculture and Biotechnology Division, Bhabha Atomic Research Centre, Mumbai, India; ^7^ Homi Bhabha National Institute, Training School Complex, Anushaktinagar, Mumbai, India

**Keywords:** lentil, disease outbreaks, conventional breeding, new breeding technologies, biotic stress, yield

## Abstract

Lentil, an important cool season food legume, is a rich source of easily digestible protein, folic acid, bio-available iron, and zinc nutrients. Lentil grows mainly as a sole crop in the winter after harvesting rice in South Asia. However, the annual productivity is low due to its slow growth during the early phase, competitive weed infestation, and disease outbreaks during the crop growth period. Disease resistance breeding has been practiced for a long time to enhance resistance to various diseases. Often the sources of resistance are available in wild crop relatives. Thus, wide hybridization and the ovule rescue technique have helped to introgress the resistance trait into cultivated lentils. Besides hybridization, induced mutagenesis contributed immensely in creating variability for disease tolerance, and several disease-resistant mutant lines have been developed. However, to overcome the limitations of traditional breeding approaches, advancement in molecular marker technologies, and genomics has helped to develop disease-resistant and climate-resilient lentil varieties with more precision and efficiency. This review describes types of diseases, disease screening methods, the role of conventional and new breeding technologies in alleviating disease-incurred damage and progress toward making lentil varieties more resilient to disease outbreaks under the shadow of climate change.

## 1 Introduction

**Figure 1:**
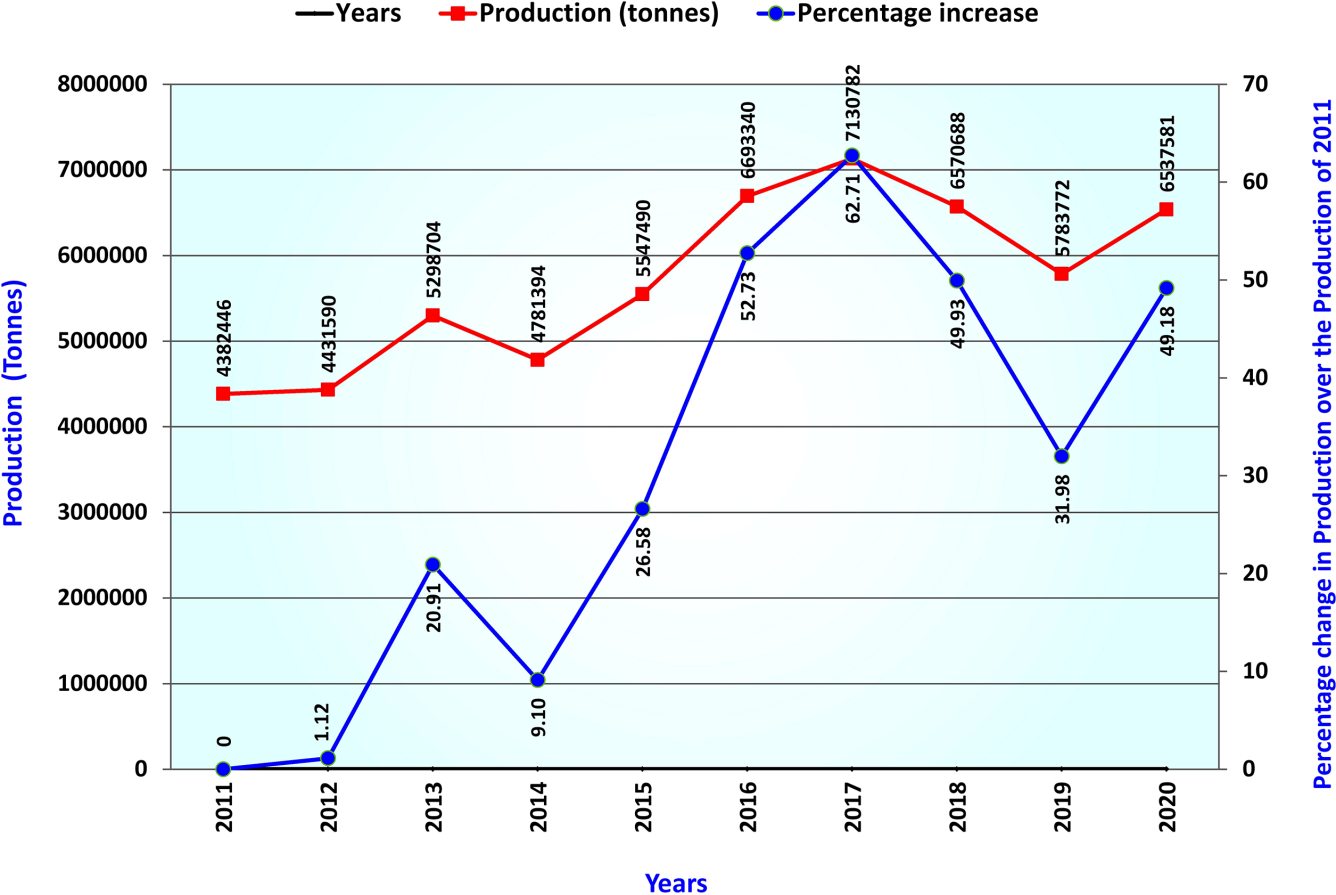
Trend of worldwide production volume of lentil over the last ten years.

Lentil (*Lens culinaris* Medik 2n=2x=14), belonging to the Fabaceae family, is one of the oldest domesticated cool season food legumes (Zohary 1999). At present the accepted name for lentil is *Vicia lens* (L.) Coss. & Germ. Lentil grains are a rich source of protein, vitamins, fiber, and micronutrients such as iron, zinc, magnesium, and folate, consumed in various raw, cooked, and processed forms (Mitchell et al., 2009; Sen Gupta et al., 2013; Joshi et al., 2017; Raina et al., 2022a). Besides, lentil enriches soil nitrogen through biological nitrogen fixation and condition soil health in long-term cereal-legume cropping sequences. The crop is cultivated over sub-tropical to temperate areas worldwide and is one of South Asia’s famous and highly consumed pulse crops (Alghamdi et al., 2014). Lentil occupies the 5^th^ position in total production among pulses worldwide and supports nutrition in low- and middle-income countries (Joshi et al., 2017; Warne et al., 2019). Worldwide lentil production has increased by 49% over the last 10 years and surpassed 6.5 million tons in the year 2020 (Yang et al., 2021; FAOSTAT, 2022) (**Figure 1**). Due to early domestication, lentil is grown as a sole pulse crop in rice fallow or *paira* crop in South Asia. It is usually grown in lower elevated land during winter, at higher altitudes during spring, and as green lentils during summer in some parts of the World beyond South Asia.

Several biotic stresses cause a huge yield loss and are emerging as threats to be addressed quickly for yield stability (Erskine et al., 1994). Early interventions of disease resistance breeding involving intra-specific hybridization has increased the average yield of lentils from 560 kg/ha to 950 kg/ha within a few decades (Singh et al., 2014). However, climate change exposes lentils to extreme weather events (drought and terminal heat), leading to increased disease outbreaks and eventually hampering yield stability (Raza et al., 2019). Among various diseases, fungal pathogens are the most threatening that reduce plant population drastically at every growth period from seedling to the pod-bearing stage. For instance, *Ascochyta* blight infection caused 30-70% yield reductions in Canada, the United States of America, Australia, and northern parts of India (Morrall and Pedersen, 1991; Singh et al., 2013a). In comparison, *Colletotrichum truncatum* caused 60% yield reductions in Canada (Morrall. 1997; Buchwaldt et al., 2013). Stemphylium blight incurs nearly 95% yield loss in India (Sinha and Singh, 1993). Further, North East India, Nepal and Bangladesh reported considerable yield loss in lentil due to Stemphyllium blight (Bakr and Ahmed, 1992). Besides yield reductions, fungal blight disease induces leaf drop, wilting, pod and seed lesions, and complete plant mortality (Taylor et al., 2007). The best way to mitigate the dreadful consequences of fungal diseases is to develop disease-resistant varieties.

Disease screening among available germplasm has not yielded desired results in identifying extremely resistant lines for Stemphyllium blight except for a few moderate resistant sources in Eastern India (Mondal et al., 2017). Uncertainty in rainfall and rise in atmospheric temperature facilitate disease outbreaks and turns some minor diseases into prominent dreadful diseases. For instance, anthracnose caused by *Colletotrichum truncatum*, is becoming a major disease in Canada (Buchwaldt et al., 2018). Thus, it is worth putting efforts into guarding the lentil crop against durable, multiple minor, and major diseases in climate-changing scenarios (Cowling, 1996). In addition, lentils possess a narrow genetic base due to their limited domestication involving very few traits that leads to very sporadic resistance to disease in cultivated gene pools (Ladizinsky, 1987; Zohary, 1989). Moreover, the quick co-evolution of pathogens causes more yield losses and demands increased genetic diversity using CWRs and new breeding lines with improved resistance (Dodds and Thrall, 2009; Tullu et al., 2006a; Singh et al., 2014). Therefore, efforts are needed to minimize the quick pace of pathogen co-evolution (Negussie et al., 2005). Still there is a opportunity for screening available germplasm for disease resistance at various environments which will provide a source materials for disease inheritance, QTL identification and gene isolation study. Therefore, artificial screening protocols are required to confirm the resistance source vis-à-vis newly identified QTLs. New plant breeding technologies like, genomics assisted breeding (GAB), genomic selection (GS) and gene editing should be painstakingly carried out for developing lentil cultivars with improved tolerance against all dreadful diseases. Recent advances in genomics, including identifying specific QTLs associated with disease tolerance and a few differentially expressed genes from the QTL region, have broadened the understanding of lentil disease resistance (Saha et al., 2010a; Cao et al., 2019). This review describes important aspects of disease resistance and the role of breeding strategies in developing disease-resistant lentil varieties.

## 2 Major diseases of lentil

**Figure 2:**
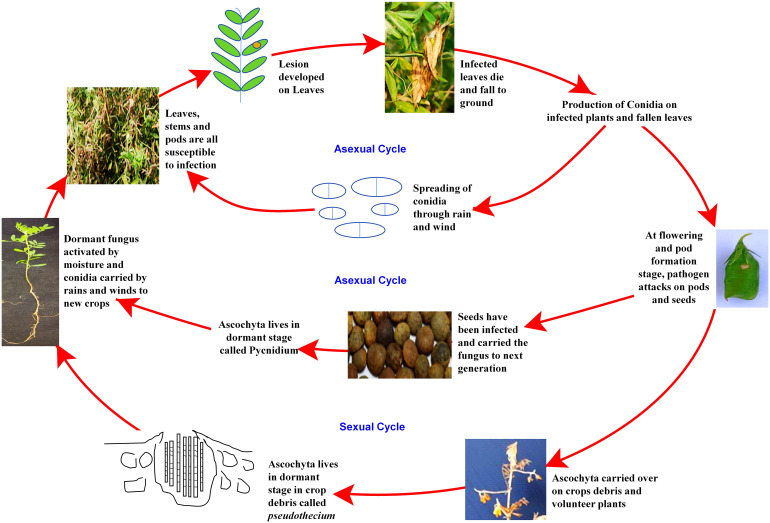
Disease cycle of Ascochyta blight disease of lentil.

**Figure 3:**
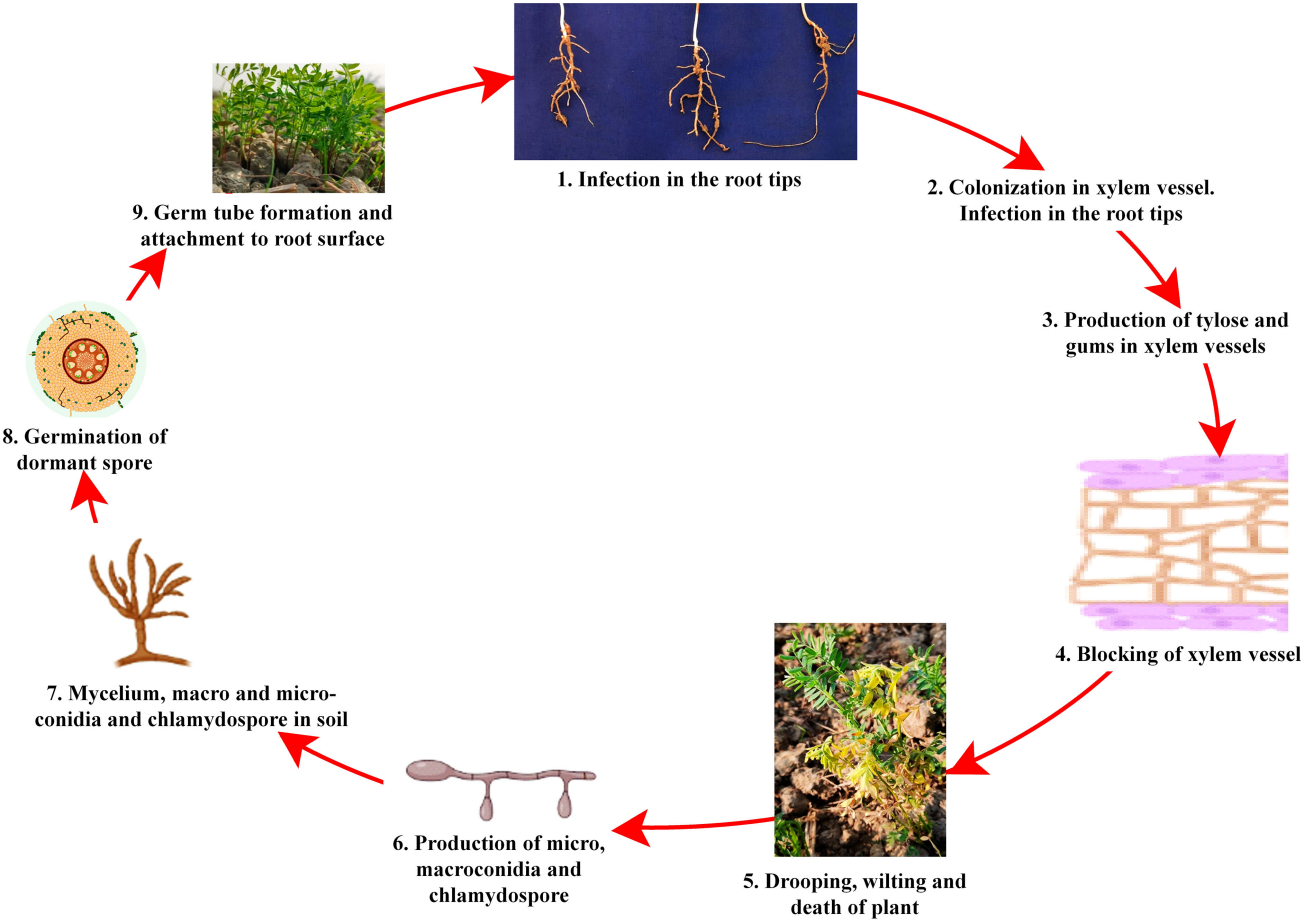
Disease cycle of Fusarium wilt disease of lentil.

Despite the high demand for lentil in South-east Asia, a declining trend in farmers’ adoption of lentil is being observed due to several biotic stresses, which limit the yielding potential. Several biotic constraints, such as fungi, bacteria, viruses, insects, nematodes, phytoplasmas, and weeds, cause a substantial reduction in average annual yield (Chen et al., 2009; Darai et al., 2017). For instance, Fusarium wilt can cause a 50 to 100% reduction in yield (Tiwari et al., 2018). However, fungal pathogens are the most dreadful and infect almost all parts, such as stems, roots, leaves, pods, and seeds, thus reducing their marketability (Bayaa et al., 1994; Bhadauria et al., 2017a). Most foliar fungal pathogen affects photosynthetic apparatus after successful colonization and sporulation, produce toxins, and cause blight (Chen et al., 2009; Chen et al., 2013). In the case of a wilt pathogen, xylem vessels get blocked and eventually restrict the upward movement of water (Erskine et al., 1994; Darai et al., 2017). Disease cycle of a foliar fungal pathogen, *Ascochyta lentis* (**Figure 2**), and a wilt pathogen, *Fusarium oxysporum* f.sp. *lentis* (**Figure 3**) represented contrasting features of these two major pathogens of lentil. The new infection of *Ascochyta* can occure through infected seed (from pycnidium) or through the resting spores (from pseudothecium) from crop debris of previous season (**Figure 2**). In case of wilt pathogen, the new infection arises from soil borne chlamydospore or micro/macro-conidia (**Figure 3**). In addition to fungal diseases, lentil production is substantially reduced by bacterial diseases such as bacterial leaf spot, bacterial root rot, bacterial blight, etc. In general, bacteria overwinter in infected seed and crop debris and sequentially infect cotyledons, leaves, and vascular system, multiply rapidly in the xylem and cause systemic infection producing stem and leaf lesions. Internally, bacteria move between cells, up or down in the vessels and ooze out through splits in the tissue and re-enter stems or leaves through stomata or wounds (Glazebrook et al., 2005). A detailed list of lentil diseases is furnished in **Table 1**.

**Table 1:** Economically important fungal and bacterial diseases and their extent of yield loss in lentil.

Name of the disease	Causative agent	Extent of yield loss
Anthracnose	*Colletotrichum truncatum* (Schw.) Andrus & Moore.	60% (Morrall. 1997; Buchwaldt et al., 2013)
Aphanomyces root rot	*Aphanomyces euteiches* C. Drechsler	80% (Gaulin et al., 2007)
Ascochyta blight	*Ascochyta lentis* Bond. &Vassil.	30 – 70% (Gossen and Morrall, 1983; Singh et al., 2013a)
Botrytis grey mould	*Botrytis cinerea* Pers. Ex Fr. and *Botrytis fabae* Sard.	50 – 100% (Haware and McDonand, 1992; Bayaa and Erskine, 1998; Davidson et al., 2004)
Collar rot	*Sclerotium rolfsii* Sacc.	Up to 50% (Asghar et al., 2018)
Fusarium wilt	*Fusarium oxysporum* f.sp. *lentis*	67 – 100% (Garkoti et al., 2013; Tiwari et al., 2018)
Lentil rust	*Uromyces viciae-fabae* (Pers.) Schroet.	60 – 69% (Sepulveda, 1985; Chen et al., 2009)
Powdery mildew	*Erysiphe trifolli*, *E. diffusa*, *E. pisi* and *Leveillula taurica* (Lév.) Arnaud.	5.5 – 15.5% (Singh et al., 2013b)
Sclerotinia rot	*Sclerotinia sclerotiorum* (Lib.) de Bary.	80% (Ahmed and Akhond, 2015)
Stemphylium blight	*Stemphylium botryosum* Wallr.	95% (Sinha and Singh, 1993)
Bacterial leaf spot	*Xanthomonas* sp.	94% (Richardson and Hollaway, 2011)
Bacterial blight	*Pseudomonas syringae* pv. *Syringae*	5% (Adhikari et al., 2018)

It is important to note that some diseases are common in almost every lentil-growing region of the world, such as Fusarium wilt and Ascochyta blight. In contrast, many are limited to areas such as Alternaria blight (restricted in India, Ethiopia, and Egypt) (Taylor et al., 2007). However, the economic importance of a disease is not necessarily characterized only by its geographical distribution. A disease with limited occurrence may still cause significant economic losses and lead to devastating effects in conducive conditions (Chen et al., 2009). The extent of yield loss in lentil as a result of different pathological diseases has been reported by several researchers (**Table 1**).

In addition to fungi and bacteria, viruses are also capable of affecting lentil productivity across the globe (Beniwal et al., 1993). About 30 virus species belonging to 16 genera, representing 9 families, with single-stranded RNA or DNA, affect lentil productivity (Chen et al., 2009). Viruses hijack the plant cell machinery, use its nucleic acids and proteins for their multiplication, and can traverse through plasmodesmata from one cell to another. At least ten viruses infect lentil in field conditions (Bos et al., 1988; Makkouk et al., 1992). Of these viruses, pea seed-borne mosaic virus (PSbMV) is more common and dreadful, decreasing the seed yield by up to 72% (Aftab et al., 1992; Kumari et al., 2009). The important viral diseases, their causal organism, and their genomic features are furnished in **Table 2**.

**Table 2:** Economically important viral diseases in lentil, causative agents, their taxonomy, genetic constitution and extent of damage.

Name of the virus	Genus	Family	Genome	Extent of damage
Alfalfa mosaic virus or AMV	*Alfamovirus*	*Bromoviridae*	(+) ssRNA	–
Bean leaf roll virus or BLRV	*Luteovirus*	*Luteoviridae*	(+) ssRNA	50 - 91%
Bean yellow mosaic virus or BYMV	*Potyvirus*	*Potyviridae*	(+) ssRNA	34 - 96%
Beet western yellows virus or BWYV	*Polerovirus*	*Luteoviridae*	(+) ssRNA	–
Broad bean stain virus or BBSV	*Comovirus*	*Comoviridae*	(+) ssRNA	14 - 61%
Cucumber mosaic virus or CMV	*Cucumovirus*	*Bromoviridae*	(+) ssRNA	75 - 84%
Faba bean necrotic yellows virus or FBNYV	*Nanovirus*	*Nanoviridae*	ssDNA	80 - 90%
Pea enation mosaic virus-1 or PEMV-1	*Enamovirus*	*Luteoviridae*	(+) ssRNA	16 - 50%
Pea seed borne mosaic virus or PSbMV	*Potyvirus*	*Potyviridae*	(+) ssRNA	23 – 73%
Pea streak virus or PeSV	*Carlavirus*	*Flexiviridae*	(+) ssRNA	–
Subterranean clover red leaf virus or SCRLV	*Luteovirus*	*Luteoviridae*	(+) ssRNA	–

Besides viruses, phytoplasma from 16SrII-C group also causes a significant loss in lentil productivity and produces symptoms like floral malformation, little leaf, chlorosis, and excessive growth of branches (Akhtar et al., 2016). However, literature is scanty on the extent of damage caused by phytoplasmas. While discussing the diseases, it is imperative to mention the role of weeds in reducing the overall productivity of lentils. By virtue of the short height and slow growth rate of lentils in the early seasons of their development, weeds outperform the crop for nutrients, light, space, and water and result in huge yield losses ranging from 20-84% (Basler, 1981; Yenish et al., 2009). Some of the crucial weeds critically reducing lentil productivity include *Avena fatua*, *Loliu multiflorum*, *Phalaris minor, Poa annua*, *Setaria viridis*, *Convolvulus arvensis*, *Cirsium arvense*, *Cuscuta campestris, C. chinensis Cynodon dactylon*, *Cyperus rotundus* and parasitic flowering plants (*Orobanche crenata*, *O. aegyptiaca*, *Phelipanche aegyptiaca*) (Rubiales et al., 2009). *Orobanche* infestations in Turkey resulted in 59% yield losses (Yolcu et al., 2020). Similarly, *Cuscuta chinensis* are dreadful weeds and can reduce the lentil productivity by 87%. Moreover, weed-borne insects, pests, and pathogens compounded adverse effects in lentils (Moorthy et al., 2003). Irrespective of the nature of devastation and causative agents, disease resistance can be improved using different genetic resources.

## 3 Genetic resources for disease resistance

Successful plant breeding depends on accessible genetic variability in the germplasm and its sustainable exploitation (Upadhyaya et al., 2011). Therefore, it is imperative to have extensive knowledge of lentil genotypes that are potential sources of disease resistance (**Table 3**). *Lens culinaris* ssp *culinaris* categorised as cultivated lentil in the genus consisting of a primary gene pool (including *Lens orientalis*) and a secondary gene pool (including *Lens nigricans*, *Lens ervoides* and *Lens odomensis*) (Muehlbauer et al., 1995). However, Fratini and Ruiz (2006) categorized *Lens ervoides* and *Lens nigricans* in the tertiary gene pool. Recently, *Lens culinaris*, *Lens tomentosus*, and *Lens orientalis* have been categorized in the primary gene pool; *Lens odemensis* and *Lens lamottei* were placed in the secondary gene pool; *Lens ervoides* was kept in the tertiary gene pool, and *Lens nigricans* were kept in the quaternary gene pool (Wong et al., 2015). Wild relatives of lentil such as *Lens culinaris* ssp *orientalis*, *Lens ervoides*, *Lens odemensis*, and *Lens nigricans* are potential and promising donors of foliar disease resistance (Bayaa et al., 1994; Ye et al., 2000). *Lens ervoides* possess genetic loci that confer partial disease resistance against Stemphylium blight (Podder et al., 2013), Fusarium wilt (Singh et al., 2017), Ascochyta blight (Tullu et al., 2010). Vail et al. (2012) also reported partial resistance to antrhacnose in cultivated lentil (*Lens culinaris*) genotypes. While, *L. ervoides* and interspecific-hybridization-derived lines showed significantly more resistance than the cultivated lentil genotypes (Vail et al., 2012). Similarly, *Lens lamottei*, *Lens ervoides* and *Lens nigricans* showed the highest resistance against anthracnose disease (Tullu et al., 2006a). However, complete resistance to Stemphylium blight has been reported in *Lens tomentosus* (Guerra-García et al., 2021). The wild species, *Lens ervoides*, *Lens nigricans* and *Lens odomensis* harbor resistance against rust, fusarium wilt and powdery mildew. In addition, *Lens culinaris* ssp *orientalis* and *Lens culinaris* ssp *tomentosus* revealed complete resistance against fusarium wilt and powdery mildew (Gupta and Sharma, 2006). A total of 58,045 accessions of lentil are maintained worldwide (FAO, 2010). National Bureau of Plant Genetic Resource (NBPGR), New Delhi, India, maintains 7712 lentil accessions, including exotic and indigenous *Lens culinaris* ssp *culinaris*. In comparison, International Centre for Agricultural Research in Dry Areas (ICARDA) maintains 14597 accessions in its gene bank (Guerra-García et al., 2021). Among the countries, maximum lentil collection is available in Syria, Australia, Iran, USA, Russia, India, Chile, Canada, and Turkey where genotypes are conserved as *ex-situ* germplasm (Malhotra et al., 2019). Despite World-wide collection, few genotypes have been used extensively in lentil breeding to improve disease resistance. For instance, foliar disease-resistant accession ILL 5588 was exploited in Australia for disease resistance breeding against Ascochyta blight (Ford et al., 1999). Later, a novel resistance source ILL 7537 was identified (Nguyen et al., 2001). Future studies should be directed to screen large number of available lentil germplasm for various disease resistance in both field based screening and artificial screening in phytotron or high throughput phenotyping facility. In developing disease-resistant cultivars, proper germplasm screening is an important step towards developing disease-resistant cultivars.

**Table 3:** Genetic resource of lentil for resistance to diseases.

Lentil Disease	Resistant Sources	Reference
Ascochyta Blight	VL Masoor 3, CDC Robin, 964a-46, ILL 7537, ILL 5588, ILL 358, ILL5684, Laird, Rajah, Masoor-93, ILL 4605, ILL 857, ILL5590, ILL 5593, ILL 5244, ILL 5725, ILL 179, ILL 195, ILL 201, ILL 5698, ILL 5700, ILL 5883, ILL 6212, ILL 2439, ILL 5562, Indianhead, 96, 507, 712, 859, 112082, 123452, 123514 and 123801	Nguyen et al., 2001; Ye et al., 2002; Tivoli et al., 2006; Sari et al., 2018; Bedasa, 2021
Lentil rust	IPL81, PL639, L4147, L4149, DPL 15, LL147, L4076, Pant Lentil 4, LH82-6, NDL92-1, Gudo (a resistant cultivar), R 186	Negussie et al., 2005; Dikshit et al., 2016; Singh et al., 2020
Wilt	JL 3, NDL92-1, P177-12, PL-639, Jawahar Lentil-1, VL Masoor 4, Pant Lentil 4, DPL-15, ILL 5883’, ‘ILL 5588’, ‘ILL 4400’ and ‘ILL 590’ Pant L 406, Pant L 4, Priya, Seri, VL507, IPL 306, IPA 98, Idleb 2, Idleb 3, Idleb 4, Ebla 1, ILL 6256, Firat 87, Syran 96, Talya 2, Rachayya, Hala, RL-13, RL-21, ILL 6468, ILL 9996,\ILL 6024, ILL 6811, ILL 7164, Arun, Maheswar bharti, L 7920 and DPL 58, PL 101, L 4076 (cultivar)	Pandya et al. (1980); Singh et al. (1994); Erskine et al. (1994); Sarker and Erskine (2002); Joshi and Maharjan (2003); El-Ashkar et al. (2004); Rahman et al. (2009); Parihar et al., 2017; Yadav et al., 2017; Singh et al., 2020; Taranam et al., 2021
Alternaria Blight	EC866132, IC267 67, IC201778	Roy et al., 2021
Anthracnose	*L. ervoides* accession: PI72847, IG72815 PI572330, PI572334, PI57233, BGE001814, PI298644, PI283604, PI477921, PI431809, PI432005, PI432033, PI432071, PI297287, PI572327. W627758 (*L. culinaris* spp. *culinaris*) PI320937, PI320952 (cv. Indianhead), PI345629, PI468901	Buchwaldt et al., 2004; Buchwaldt et al., 2018; Barilli et al. (2020)
Resistant to Blight, rust and Viral disease	66013-6	Hussain et al., 2008
Resistant to Stemphylium blight	LL 1370, VL 151, LL 1375, RLG 195, L 4727, L 4769, LL 1397, DL 14-2, VL 526, VL 126, RKL 14-20, IPL 334, L 4710, PL 210, PRECOZ (RC), RL-13, RL-21, ILL 6468, ILL 9996,\ILL 6024, ILL 6811, ILL 7164, Arun, Maheswar bharti	Mondal et al., 2017; Yadav et al., 2017

## 4 Screening methodologies for disease resistance breeding

Establishing suitable and effective screening techniques is the major component of the breeding programs for disease resistance. A complete understanding of resistance type, pathogenicity, virulence pattern, and the effective breeding strategy is required to obtain desirable results. A sufficient amount of research work has been carried out in the last decade to delineate the nature and durability of resistance, and effective methods of screening for resistance to several pathogens have been devised (Tullu et al., 2003; Negussie et al., 2005; Stoilova and Chavdarov, 2006; Podder et al., 2013). The following paragraphs briefly explain some of the commonly used screening methods for various diseases.

### 4.1 Screening in a natural field condition

Screening genotypes in the field condition under natural disease epidemics for selecting resistant genotypes requires an extensive knowledge of the disease epidemics and ‘hot-spots’. Diseases have different hotspots based on their congenial growth conditions. The test genotypes are grown in the hotspot regions and screened for the target disease. While screening the genotypes, some known resistant and susceptible cultivars are also planted as checks under the same environmental conditions (Ye et al., 2002). However, this approach suffers a major drawback due to its dependence upon the epidemic year for the screening and thus reduces the breeding progress. Besides, it is also dependent upon the severity of disease infestation (Porta-Puglia et al., 1994). Therefore, screening and selection of resistant genotypes may be performed under artificial conditions to achieve reliable outcomes (Ye et al., 2002). Using the field screening teachnique, Buchwaldt et al. (2004) identified 16 lentil germplam which were resistant to the antracnose disease caused by isolate Ct1. But no accessions were found resistant to isolate Ct0. Bedasa (2021) conducted field screening for Aschochyta blight in hot spot condidtion of Alemtena and Minjar (Ethiopia) and identified eight resistant lentil genotypes which showed resistant to moderate resistant reactions in both seasons.

### 4.2 Screening in the field through artificial infections

Screening of disease-resistant genotypes under artificial conditions could overcome major limitations that are frequently encountered in the natural screening method. Artificial epidemics for a particular disease can be generated in the field by using the following three methods viz., preparation of inoculums in the laboratory and application in the individual plant, scattering the diseased plant debris throughout the experimental field, and inter-planting of susceptible genotypes (spreader rows) after every 6-8 rows of test genotypes to increase pathogen populations over the field (Ye et al., 2002). Regular irrigation through flooding or sprinkler may be provided to generate the optimum relative humidity in the field (Ahmed and Morrall, 1996). Inoculums may be applied in plants by spraying the suspension (for foliar pathogen), mixing the pure culture of pathogens into the planting soil (soil-borne pathogen) or by leaf clipping method (Stoilova and Chavdarov, 2006). Methods of preparation of inoculums for artificial infection may vary significantly according to the nature of pathogen. After the inoculation, proper conditions will be required to facilitate the pathogen growth, multiplication, and disease progression. The chances of disease severity will be considerably less in the absence of optimum conditions. Furthermore, inter-planting susceptible genotypes with test genotypes may be a viable and most feasible option for screening disease resistance. To date, most of the resistant genotypes released were identified through artificial infection at field method. The effectiveness of this method may be affected by the interaction between genotype and environment, physiological age of the tested plant and tissue-specific expression of disease resistance (Ahmed and Morrall, 1996). Dikshit et al. (2016) screened a RIL population of lentil to screen for rust resistance by following a spreader row technique in field and successfully phenotyped the population towards identification linked molecular markers for rust resistance. Almost one-third of screened F2 population (119 plants) was found resistant to rust disease.

### 4.3 Screening in the glasshouse/greenhouse through artificial infections

Screening of genotypes for any disease resistance may be performed in a glasshouse under controlled environmental conditions. Optimum photoperiod, relative humidity, and temperature may be easily adjusted in the controlled glasshouse/greenhouse according to the requirements for disease progression. These parameters may differ as per the nature of pathogens. Using this method, test genotypes are planted under a glasshouse along with the susceptible genotypes, followed by artificial infections. Artificial infection-based screening at glasshouse could overcome major limitations of field screening. Following are the major advantages in this method, i) disease screening may be performed in off-season and at any developmental phenophase, ii) manipulation of environmental conditions can be accustomed easily for proper disease development, iii) interference from other biological agents can be avoided by creating clean environments, iv) the inoculums can be more evenly distributed and consequently reduce the chance of escapes (Ye et al., 2002; Porta-Puglia et al., 1994). Looking at the merits of this method, it may be best suited for screening disease-resistant genotypes and understanding the genetic mechanism of disease resistance. This method is also suitable for screening genotypes with novel resistance genes for new virulent strains using a range of pathotypes or isolates. However, this method is quite costly and may not be useful for screening large size of segregating population. For soil borne disease like Fusarium wilt, sick plot techniques in field used for the identification of resistant germplasm (Bayaa and Erskine, 1990; Bayaa et al., 1995; Bayaa et al., 1997; Eujayl et al., 1998). The resistance against vascular wilt caused by *Fusarium oxysporum* f.sp. *lentis* Vasud. & Srin, was screened in a sick plot technique in a polyhouse using artificial inoculation of a Syrian isolate of this fungus at the seedling stage (Bayaa et al., 1995). Three accessions each of *Lens culinaris ssp. orientalis* and *L. nigricans* ssp. *nigricans* and two of *L. nigricans ssp. ervoides* were found to possess resistance at the reproductive growth stage. Further, three accessions (ILWL 79 & ILWL 113 of *L. culinaris ssp. orientalis* and ILWL 138 of *L. nigricans ssp. ervoides*) were tolerant. Negussie et al. (2005) identified Gudo and R-186 as sources of rust resistance based on glasshouse screening. While, Fiala et al. (2009) conducted experiment to screen a segregating population for anthracnose resistance under controlled conditions in a Conviron growth chamber (Model GR178; Winnipeg, MB) maintained at 21°C day and 18°C night temperatures with an 18-h photoperiod under fluorescent and incandescent lighting with artificial inoculation Ct0 and Ct1 isolates. They found 103 F_5:6_ RILS were resistant to Ct0 isolates, while only 19 were resistant to Ct1 isolate.

### 4.4 Screening of disease resistance in laboratory

When creating disease epiphytotics is difficult in field and greenhouse conditions, then some laboratory based screening methods like detached leaf test (Hanounik and Maliha, 1986); culture filtrates or purified phytotoxins based selection method (Buiatti and Ingram, 1991) and cut-twig method (Sharma et al., 1995) may be performed to assess host reactions (Porta-Puglia et al., 1994). Hanounik and Robertson (1988) employed a detached leaf test to evaluate disease resistance against chocolate spots in faba bean and concluded that this method could easily be followed for foliar disease resistance screening in a laboratory environment. In detached leaf test, fully expanded leaflets of a similar age were detached from the fifth node position of test plants and susceptible check plants. These leaflets were laid flat on a 2 cm thick moist sponge lining the bottoms of 90 X 40 X 5 cm galvanized metal pans, then inoculated separately with fungal spores (around 0.1 ml suspension containing 600,000 spores). One droplet was placed on each half of the upper lamina surface of each leaflet, then the pans were covered immediately and incubated at room temperature for disease development. Sharma et al. (1995) used the ‘cut-twig’ technique to screen resistance against *Ascochyta rabiei* in chickpea. This method includes inoculation of spores in single cut branches with spores. Culture filtrates or purified phytotoxins based selection method was used first by Carlson in 1973 using haploid cell lines of *Nicotiana tabacum* (Carlson, 1973). It was suggested that some purified phytotoxins positively correlate with plant tolerance and resistance behavior to pathogens. Therefore, this method has been used to screen genotypes for various disease resistance. However, the results sometimes seem to be contradictory, and there were systems where such a correlation seems to be proven only for some cultivars and not in other cultivars (Buiatti and Ingram, 1991; Buiatti and Scala, 1984; Buiatti et al., 1985; Kono, 1989). Therefore this method was not used regularly by the breeders for screening purposes. Laboratory testing is instrumental in selecting a resistant plant in earlier generations when the number of seeds per line is limited. Since disease reaction can be confirmed using twigs/leaf (with petiole)/branch, the entire plant is kept aside for seed production and further multiplication. However, there is a lack of research regarding the possible use of such methods in food legumes like lentil.

### 4.5 Screening of disease resistance genes using molecular markers

With the advent of molecular markers and next-generation sequencing approaches, it became easy to identify the genes/QTLs associated with specific disease resistance. The linkage between genes and molecular markers may be accurately calculated with the help of recent genomic approaches, *viz.* bi-parental QTL mapping (Collard et al., 2005), association mapping (Chakraborty and Weiss, 1988; Kruglyak, 1999; Yu et al., 2006), QTLSeq (Takagi et al., 2013). Development of molecular markers include generation of mapping population, screening of polymorphic markers, phenotyping of the mapping population, genotyping of population with polymorphic markers, generation of linkage map and QTL analysis and validation of linked markers. A molecular marker tightly linked with the gene of interest/QTL may be used to screen a large number of segregating populations with the minimum phenotyping in the field. This is relatively effortless, feasible, and much more reliable than other methods. However, disease-resistant genes’ penetrance and expressivity may vary in genotypes and environmental conditions. Furthermore, the results may be confirmed by field screening due to the occurrence of recombination event between resistance gene and marker loci. Tar'an et al. (2003) screened a recombinant inbred line (RIL) population using markers linked to *ral1* (for ascochyta blight), *AbR1* (for ascochyta blight) and to the major gene for resistance to anthracnose using molecular markers UBC2271290, RB18680 and OPO61250, respectively and confirmed pyramiding of resistance genes for both Ascochyta blight and Anthracnose disease in 11 RILs. There are many more examples of such available markers for different diseases of lentil (**Table 4**). All these markers have potential to screen the segregating populations towards resistant genotypes identification. Moreover, these markers will help to pyramid multiple resistance genes in a agronomically superior lentil variety.

**Table 4:** Details of QTLs and genes identified and mapped for disease resistance in lentil.

S. No.	Trait	Type of marker	Marker name/QTLs/Genes	Mapping Populations	Phenotypic variationExplained by the QTL (%)	References
1.	Resistance to *Ascochyta* blight	RAPD	*RV01–RB18*	ILL5588 × ILL6002	90	Ford et al. (1999)
		RAPD, SCAR	UBC227_1290_ and OPD-10_870_ for *ral 2* gene	Eston x Indian head	–	Chowdhury et al. (2001)
		RAPD, ISSR	OPB18_680_ OPV1_800_	ILL5588 x L692-16-1	29 – 36	Tar’an et al. (2002)
		RAPD	UBC227_1290_ for *ral1* gene, RB18_680_ for *AbR1* gene.	CDC Robin x 964a-46	–	Tar'an et al. (2003)
		RAPD, AFLP, and ISSR	Five QTLs on LG1, LG2, LG4 LG5.	ILL5588 × ILL7537,	7 – 69	Rubeena et al. (2006)
		RAPD, AFLP, and ISSR	Four QTLs on LGI and LG II.	ILL7537 × ILL6002	6 – 34	Rubeena et al. (2006)
		AFLP and RAPD	ctcaccB *and LCt2*	Eston × PI320937	41	Tullu et al. (2006b)
		EST-SSR/SSR	DK 225–UBC825c	North Weld (ILL5588) × Digger (ILL5722)	61	Gupta et al. (2012)
		SNP, SSR	Two major QTLs on LG1 and LG 2. LcC12416p463 and LcC03040p469 are the SNP markers for respective QTL	CDC Robin × 964a-46	–	Sari (2014)
		SNP and SSR	Three QTLs: AB_IH1, AB_IH2.1 & AB_NF1. Markers: SNP20005010, SNP20002370, SNP20001370, and SNP20001765	Indianhead × Northfield	7 – 47	Sudheesh et al. (2016)
		SNP and SSR	Two QTLs: AB_IH1 & AB_IH2.2 Marker: SNP20005010	Indianhead × Digger	22 - 30	Sudheesh et al. (2016)
		SNPs and short InDels	AS-Q1, AS-Q2, and AS-Q3	*L. culinaris* (Alpo) x *L. odemensis* (ILWL235)	28.46	Polanco et al. (2019)
2.	Resistance to Stemphylium blight	SSR, SRAP, RAPD	QLG4_80_, QLG2_49_, QLG3_3_, QLG4_81_ Markers: ME4XR16c, MR5XR10, and UBC34	ILL6002 × ILL5888	25 - 46	Saha et al. (2010a)
		SNP	qSB-2.1, qSB-2.2, qSB3 Markers: Contig271180p29128, Contig313227p47568, Contig406212p17766	L01-827A x IG 72815	9.9 -18.30	Bhadauria et al. (2017a)
3.	Resistance to Lentil Rust	SSR and SRAP	GLLC527 (SSR)	PL8 x L4149	–	Dikshit et al. (2016)
		SSR	GLLC106	FLIP-2004-7L x L-9-12	–	Fikru et al. (2016)
		SRAP	F7XEM4a	ILL-4605 x ILL-5888.		Saha et al. (2010b)
4.	Resistance to Anthracnose	RAPD, AFLP	*LCt-2 locus* Markers: OPEO6(1250), UBC-704(700), EMCTTACA(350), EMCTTAGG(375), EMCTAAAG(175)	Eston lentil x PI 320937	–	Tullu et al. (2003); Tullu et al. (2006a)
		RAPD	OPO6_1250_	CDC Robin x 964a-46	–	Tar'an et al. (2003)
		SNP	qANTH0-3, qANTH0-5.1 and qANTH0-5.2 for race *Ct0* qANTH1-3.2, qANTH1-5.1 and qANTH1-5.2 for race *Ct1*	L01-827A x IG 72815	47.58 for Ct0 and 54.82 for Ct1	Bhadauria et al. (2017a)
		RNA Seq.	*-*	LR-66-528 x LR-66-524	–	Bawa (2020)
5.	Resistance to Fusarium wilt	RAPD	*Fw locus* Markers: OPK-15_900_, OP-BH_800_ and OP-DI5_500,_ OP-C04_650_	ILL5588 x L692–16‐l(s)	–	Eujayl et al. (1998)
		AFLP	p17m30710	ILL5588 x L 692-16-1(s)	–	Hamwieh et al. (2005)
		SSR	SSR59-2B	ILL5588 x L 692-16-1(s)	–	Hamwieh et al. (2005)
6.	Resistance to Aphanomyces Root Rot	SNP (GBS)	Q.RRI-Lc2.1, Q.BLU-Lc2.1, Q.SAT-Lc2.1, Q.CAN-Lc2.1, Q.AGI-Lc2.1, Q.RRI-Lc5.1 and Q.AGI-Lc5.1	K192-1 x K191-2	5.2 – 12.1	Ma et al. (2020)
		SNP (GBS) Association mapping	G.RRI-Lc1.1 and G.BLU-Lc1.1, G.RDL-Lc4.1 and G.RPL-Lc4.2, G.RRI-Lc5.1 and G.SAT-Lc5.1	326 accessions (AM)	1.4- 21.4	Ma et al. (2020)

## 5 Resistance breeding in lentil

Reducing the pathogen entry at the initial phase of infection is the basic strategy for inhibiting disease progress (Nene et al., 1998). It has been stated that open canopy architecture is less sensitive to foliar diseases than the closed canopy. Hence, breeding for the canopy architecture in lentils will indirectly provide resistance to biotic stresses (Pedersen and Morrall, 1994). Similarly, leafless branches in pea tolerated lodging and were less prone to foliar diseases (Heath and Hebbelthwaite, 1985). A similar strategy, improving the harvest index, can be followed in selecting disease-resistant lentil genotypes. Epidemiology of disease is very important to decide the breeding strategies to be followed in field. For instance, *Aschochyta* blight heavily infests lentil during cool and wet weather conditions and infection frequency reaches a maximum at 10-15°C (Pedersen and Morrall, 1994; Nene et al., 1998). Artificial infection in the field, glasshouse and laboratory are initial and important steps in screening true resistance for breeding programme and developing standard off-season disease-specific screening protocols (Ye et al., 2002). ICARDA led multilocational disease screening throughout the centres around the globe has facilitated the registration of disease-resistant cultivars in many countries (Russell, 1994; Singh et al., 1994; Erskine et al., 1996). A small seeded *Lens culinaris* variety ‘Pant Lentil 4’ was developed through pedigree selection in a 3-way cross (UPL175 × (Pant L 184 × P288)) in the North-Western plains of India. This variety has higher seed yield and resistance to rust, wilt and Ascochyta blight (Singh et al., 1994). In the last 15 years, about 38 disease resistant/tolerant lentil varieties were developed through recombination breeding technique in India that were either released by central varietal release committee or state variety release committee (Project Coordinator’s Report, Annual Group Meet on MULLaRP, AICRP, ICAR, IIPR, Kanpur 2017-18; https://www.seednet.gov.in ). The pedigree method developed a multiple disease-resistant variety ‘Debine’ in Ethiopia recently. This variety had comparable resistance/tolerant levels to major lentil diseases such as Aschocyta blight, rust, and root rot (Tekalign et al., 2022). The bulk method is ideal for applying natural selection for disease resistance in segregating populations. While early generation selection using disease nursery or the creation of artificial disease epidemic will be good for pedigree breeding methods. The combined bulk and pedigree method has been used for a long time in lentil resistance breeding (Singh, 1993; Muehlbauer et al., 1995). Hybrid plants (*Lens culinaris* x *Lens ervoides*) with improved disease resistance have been developed using embryo culture techniques (Ladizinsky et al., 1985). Further, the rearrangement of resistant alleles through chromosome translocation and recombination has developed novel resistance against a fungal disease that originated from *Lens ervoides* (Bhadauria et al., 2017a). Recently, successful gene introgression from wild lentil *L. ervoides* was evident in an advanced backcross population that showed significant variation in anthracnose and Stemphylium blight disease resistance and held a promise to provide valuable disease-resistant genetic stocks in a future breeding program (Gela et al., 2021). Simple crossing involving multiple resistance sources can lead to the study of the complex inheritance of a particular disease. The RIL population among contrasting parents can deliver information regarding the distribution of disease reactions in a defined population. Gene pyramiding can be used to accumulate such multiple disease resistance in a single genotype through marker-assisted selection. Barilli et al. (2020) proposed using moderate to highly resistant germplasm of diverse origin as donors for Aschochyta blight resistance to broaden the genetic diversity of the evolved resistant genotypes. Interspecific hybridization among newly identified resistant lentil species is very important to develop pre-breeding materials for disease resistance breeding. To maintain the genetic base of the crop and reduce its genetic erosion, the classical breeding approach must be continued to develop the genotypes with new gene combinations. Till now, interspecific and intraspecific hybridization in lentil has eveloved many resistant recombinants. These resistant recombinants/breeding lines will be of great use in transferring resistance into well adapted varieties. Efficient selection in such process requires linked molecular markers that will help in pyramiding diverse resistant alleles in a superior variety.

## 6 Marker-assisted breeding for disease resistance

Identifying and mapping genes/QTLs controlling the desired phenotype is the basic and important step in marker-assisted breeding for crop improvement. Among various genomic resources, molecular markers have played a significant role in speeding up crop improvement and understanding the genetic basis of economically important traits (Varshney and Tuberosa, 2007). The availability of polymorphic markers and genetic linkage maps makes it easier to identify and map the QTLs for a trait of interest through family-based linkage mapping or germplasm-based association mapping approaches (Mackay and Powell 2007). Linkage-based QTL identification and mapping require a properly developed experimental population with a suitable size, developed from two contrasting parents (Bohra et al., 2014; Mitchell-Olds 2010). However, association mapping or linkage disequilibrium mapping requires a set of genetically diverse genotypes, landraces, or natural populations (Mackay and Powell 2007). Linkage and association-based QTL mapping follows the principles of the forward genetic approach and hence depend on phenotypic expressions or variations available in the experimental population for the trait of interest.

Most of the researchers followed the identification and mapping of QTL through a linkage-based approach for economically important traits. Several QTLs have been identified and mapped for agronomic traits (days to flowering, plant height, seed size, pod dehiscence, winter hardiness, growth habit, seed yield), disease resistance (ascochyta blight, stemphylium blight, rust, anthracnose, fusarium wilt and aphanomyces root rot) and abiotic stress tolerance (boron tolerance) by utilizing both inter- and intraspecific maps (Ford et al., 1999; Rubeena et al., 2006; Tullu et al., 2008; Saha et al., 2010a; Bohra et al., 2014; Dikshit et al., 2016; Sudheesh et al., 2016; Bhadauria et al., 2017a; Polanco et al., 2019). A comprehensive list of identified QTLs for resistance to Ascochyta blight, Stemphylium blight, rust, anthracnose, Fusarium wilt, and Aphanomyces root rot in lentil is presented in (**Table 4**). Saha et al. (2010a) employed SSR, SRAP, and RAPD markers to identify QTL (*QLG480–81*) for Stemphylium blight resistance. In contrast, Bhadauria et al. (2017a) identified two QTLs (*qSB-2.1 and qSB-2.2)* for resistance to Stemphylium blight using SNP markers. Three QTLs viz., *F7XEM4a*, *GLLC527* and *GLLC106* conferring resistance to lentil rust were identified by Saha et al. (2010b); Dikshit et al. (2016), and Fikru et al. (2016), respectively using SSR markers (**Table 4**). Substantial research in resistance to anthracnose is also evident in the number of identified QTLs viz., *OPO61250* (Tar'an et al., 2003), *LCt-2* (Tullu et al., 2003), *qANTH1.2-1, ANTH1.2-2* and *qANTH1.3-2* (Bhadauria et al., 2017b) in lentil. Besides anthracnose, Eujayl et al. (1998) identified one QTL (*fw1*) using the RAPD marker, while Hamwieh et al. (2005) identified two QTLs viz., *p17m30710* using AFLP and *SSR59-2B* using SSR markers for resistance to Fusarium wilt.

However, very few reports are available for identifying QTLs for disease resistance through association mapping. Identification of QTLs through linkage-based mapping is not as much robust as association mapping due to some limitations *viz.*, lack of high resolution, inefficiency, and requiring a long time to develop a bi-parental population (Parisseaux and Bernardo, 2004). Moreover, bi-parental mapping approach also had some inherent genetic constraints like, moderate to high segregation distortion and non-universality of linked marker reaction to other inter-specific/intra-specific populations. Alternatively, association mapping can potentially address these limitations of bi-parental linkage mapping. Association analysis may identify QTLs further through high-resolution mapping using historical recombination available in diverse genotypes or natural populations (Mackay and Powell 2007; Ma et al., 2020). A well-designed set of association panels represented by a global mini-core collection of lentil with a high amount of genetic variation may save time and cost while performing marker-assisted breeding in this crop. Genome-wide association study (GWAS) was first demonstrated in lentil to reveal marker-trait association for Aphanomyces root rot resistance (Ma et al., 2020). Later, GWAS was used for identification of marker for improtant agronomic traits (Rajendran et al., 2021), prebiotic carbohydrates (Johnson et al., 2021) and salt tolerance (Dissanayake et al., 2021).

Molecular markers linked to desirable genes/QTL affecting a phenotype are being used now to introgress that QTL in the genetic background of improved genotypes using marker-assisted breeding (Collard et al., 2005). Several tightly linked markers (<5 cM) with high phenotypic effect are now available in lentil that may be used in marker-assisted breeding (MAB), marker-assisted backcrossing (MABC), marker-assisted gene pyramiding, marker-assisted recurrent selection (MARS), and genome-wide selection (GWS) (ana et al., 2019). Pyramiding of multiple QTLs/genes may be conducted through the multiple parent crossing, backcrossing, and recurrent selection. Pyramiding three or four genes can be achieved through three-way, four-way, or double-crossing. Ta’ran et al. (2003) identified two QTLs *viz.*, *ral1* (UBC2271290) and *AbR1* (RB18680) for Ascochyta blight resistance and one QTL (OPO61250) for resistance to anthracnose using RAPD marker. While integrating these QTLs into a single genotype through marker-assisted breeding, they found 11 RILs with all three genes. These pyramided genes explained about 55% contribution to resistance to Ascochyta blight and anthracnose. With this work’s help, they could develop a durable variety of lentils. Availibility of genomic resources in lentil will help breeders to fine map each disease resistance locus and develop candidate gene-based markers in lentil for efficient selection in future.

## 7 Role of mutation breeding in lentil improvement

Even though genomics-assisted breeding is popular in other legumes, its pace is slow in lentil due to its large genome size, narrow genetic base, low-density genetic linkage map, and difficulty in identifying beneficial alleles (Kumar et al., 2015). However, these limitations or genetic bottlenecks can be overcome by mutation breeding in popular lentil cultivars (Erskine et al., 1998). Molecular tools have infrequently been used to realize the genetic basis of a few traits related to biotic (ascochyta blight, anthracnose, rust, fusarium wilt, Stemphylium blight) and abiotic (drought, frost, cold, boron, salinity) stresses (Kumar et al., 2014). Further use of hybridization for crop improvement in lentils is limited due to its tiny flower, flower drop, low seed set in interspecific hybridization, and unease in tissue culture-based embryo rescue technique. In such inherent constraints, the narrow genetic base could be broadened using induced mutation breeding, a coherent tool for increasing genetic variability (Laskar et al., 2015; Rana and Solanki, 2015; Khursheed et al., 2018; Shahwar et al., 2019; Raina et al., 2022b; Raina et al., 2022c).

### 7.1 Types of mutants and mutant varieties of lentil for disease resistance

Lentil is responsive to both chemical and physical mutagens indicating the scope of improvement using mutation breeding (Sharma and Sharma, 1979; Gaikwad and Kothekar, 2004; Solanki and Phogat, 2005; Solanki et al., 2007). Mutation breeding was considered in lentils to improve several agronomical traits (Tyagi and Gupta, 1991; Ali and Shaikh, 2007; Ali et al., 2010; Tabti et al., 2018a), herbicide tolerance (Rizwan et al., 2017; McMurray et al., 2019), fascinating fertile mutants (Tyagi and Gupta, 1991), early maturing and dwarf mutants (Sinha, 1988; Sinha, 1989; Solanki, 2005; Solanki and Phogat, 2005), disease resistance (Bravo, 1983; http://mvgs.iaea.org , MVD, 2020) and yield (Ali and Shaikh, 2007; Ali et al., 2010). Laskar et al., 2017 and Laskar et al., 2018a; Laskar et al., 2018b) developed lentil mutant lines with improved yield and nutrient density using gamma rays and hydrazine hydrates. Mutagenic lentil populations developed at ICARDA through the treatment of ethyl methane sulfonate has shown the promise in isolation of pod shattering, herbicide tolerance and *Orobanche* tolerance (Kumar et al., 2015). Chemical mutagens mostly react with nucleotide base and modified it. This modified base impairs in base pairing and thus causes base substitution. Physical mutagen like gamma rays also causes base substitutions due to the base damage by free radicals along with its direct action in single and/or double strand break in DNA that leads to deletions, insertions, inversions, and translocations. Punia et al. (2014) reported hypervariable spontaneous generation of mutation for earliness, seed coat colour and seed size in a commercial population of lentil cultivar DPL-62. However, the frequency of spontaneous mutations is not adequate to meet the needs for genetic improvement and necessitates the use of induced mutations. Occasionally such behaviour is explained due to the activity of transposable elements (Gowda et al., 1996). It is reported that genomic shock/stress (ionizing radiations, base-damaging chemicals) induces the transposition of mobile genetic elements and causes an indirect mutation in plants (Koturbash, 2017). Success story towards induction of disease-resistant lentil variety ‘NIAB MASOOR 2002’ through gamma rays mutagenesis is well documented in mutant variety database (https://mvd.iaea.org/#!Variety/3379). Another successful example of induction of a high-yielding variety with multiple disease resistance (Ascochyta blight, rust, and Botrytis grey mould) is ‘NIAB MASOOR 2006’ obtained from 200 Gy gamma rays treatment of ILL 2580 in Pakistan (Sadiq et al., 2008). Mutation breeding in lentils is tilted more towards enhancing tolerance to biotic stress rather than direct yield improvement. Till now, a total of 18 mutant varieties have been developed in lentil crop (http://mvgs.iaea.org ) **(** **Table 5** **).** Of which, nine lentil mutants were resistant to various diseases. For instance, mutant lines viz., Binamasur-1, Binamasur-2, Binamasur-3 and NIAB Masoor-2006 are resistant to rust and ascochyta blight. Zomista and Mutant 17 MM are resistant to anthracnose and viral diseases. Another mutant, Djudje, is resistant to Fusarium wilt and Botrytis grey mould diseases (http://mvgs.iaea.org , MVD, 2020). In addition to appropriate plant material and an optimum mutagen dose, a large M_2_ population is also important for achieving success in the mutation breeding program. The success of mutation breeding in developing mutant varieties with improved yield, grain quality, and tolerance to biotic and abiotic stress is determined by factors like the genetic background of parents, the dose of mutagen, mutagenized plant population, selection criteria, successive handling of advanced mutant generation.

**Table 5:** List of disease resistant lentil mutants developed and registered under the Joint FAO/IAEA Database of Mutant Variety and Genetic Stock (http://mvgs.iaea.org ).

	Variety Name	Parent name	Mutagen	Dose	Local/National Registration Year	Character Improvement Details	Institute
1	Binamasur-1	L-5 (local genotype)	Extract of Dhatura seeds	NA	2001	High yield, tolerant to rust and blight, black seed coat	Bangladesh Institute of Nuclear Agriculture (BINA) & Bangladesh Agriculture University (BAU), Bangladesh
2	Binamasur-2	Utfala (local genotype)	Gamma rays	200 Gy	2005	High yield, early maturity, tolerant to rust and blight	BINA, Bangladesh
3	Binamasur-3	L-5 (local genotype)	EMS	0.50%	2005	High yield, early maturity, rust and blight tolerance	BINA, Bangladesh
4	Djudje	Tadjikskaya 95	Gamma rays	30 Gy	2000	High yield, dwarf bushy habit, suitable for mechanized harvesting, non-shattering, resistance to fusarium and botrytis, high protein content (27.9%), good culinary and organoleptic quality	Dobrudzha Agricultural Institute (DAI), General Toshevo, Bulgaria
5	Elitsa	Tadjikskaya 95	Gamma rays	40 Gy	2001	High yield (34.4%) and resistance to the major disease	DAI, General Toshevo, Bulgaria
6	Mutant 17 MM	NA	NA	NA	1999	Vigorous growth habit, large leaflet, pods and seeds, resistance to anthracnose, stemohylium and viruses, high yield, drought tolerance and improved cooking quality	DAI, General Toshevo, Bulgaria
7	NIAB MASOOR 2002	NA	Gamma rays	NA	2002	Erect growth habit, early maturity (120 days), black seed coat color, high grain yield, diseases recsistance and synchronous pod maturity	Nuclear Institute for Agriculture and Biology (NIAB), Faisalabad, Pakistan
8	NIAB MASOOR-2006	ILL-2580	Gamma rays	200 Gy		Higher number of pods, resistance to lodging and resistance to blight and rust	NIAB, Faisalabad, Pakistan
9	Zornitsa	Tadjikskaya 95	EMS	0.10%	2000	High yield, high protein content (28.7%), good culinary and organoleptic quality, resistance to anthracnose, viruses and ascochyta blight	DAI, General Toshevo, Bulgaria

### 7.2 Selection of genotypes and dose determination

In mutation breeding programs, selecting appropriate genotypes usually well-adapted farmer’s preferred variety, is important for the genetic improvement of existing lentil cultivars (Laskar et al., 2018a). Besides, a traditional landrace suitable for cultivation in a particular agroclimatic condition is also preferred to improve yield and quality traits. Moreover, an interspecific derivative line that may still have linkage drag can also be used as source material for further improvement. The tight linkage between the disease resistance and undesirable traits in interspecific-cross derived lines can easily be broken down using mutagens such as gamma rays, electron beams, charged particles, and fast neutrons (Joshi et al., 2020). After the selection of source material, it is recommended to study the dose-response of the particular genotypes for the evaluation of GR30 and GR50 values following probit analysis. It is always recommended to use an optimum dose that lies between GR30 and GR50 values to achieve the highest frequency of mutation and less biological damage. Combinations of physical and chemical mutagens have also been employed in the genetic improvement of lentil cultivars (Laskar and Khan, 2017). A study revealed that 0.4% of hydrazine hydrates and 400 Gy of gamma rays were maximum non-lethal strength of respective mutagens for mutation induction in lentils (Laskar et al., 2017). In contrast, lower concentrations of ethyl methanesulfonate (0.1 and 0.2%), hydrazine hydrate (0.02 and 0.03%), and sodium azide (0.01 and 0.02%) were used to develop a large mutagenized population of lentil for screening tolerant mutant for herbicide (Rizwan et al., 2017). A lower dose of gamma rays 100 Gy on cv. Idlib-3 (ILL6994) effectively generated significant variability for most lentil quantitative traits (Tabti et al., 2018b). For most of the seed propagating crops, pure seed (nucleus seed) was used as a source material for treatment with mutagens. Various factors are responsible for optimum dose determination of seeds. For gamma rays, initial moisture content and oxygenated environment are very crucial to get optimum DNA damage in seeds. For chemical mutagens, pre-treatment, types of buffer, time of treatment, cell cycle stages, and temperature are the major determinants for determining the concentration of chemicals used for mutation breeding experiments.

### 7.3 Mutant population development

Mutations are random events induced at a very low frequency and further reduced by plant recovery mechanisms. To effectively screen a desired mutant, a large-sized mutagenized population developed by using an optimum mutagen dose is required (Raina et al., 2020). Moreover, the cytotoxic effect of higher mutagen dose leads to the mortality of M_1_ plants and ultimately results in lower M_2_ population size (Goyal et al., 2021). On the contrary, a lower mutagen dose is not enough to induce a mutation and results in the progression of wild-type progeny. Thus, prior to the mutation breeding experiment, optimization of mutagen dose must be carried out using above-mentioned methods. Further a large-sized mutagenized population is recommended to screen the desired lentil mutants effectively. In case of rice, a small mutagenized population (with 10000 plants) can saturate the genome with mutations (Viana et al., 2019). Lentil possesses nearly ten times bigger genome size than rice (4063 Mbp); therefore, it requires a large mutagenized population (with at least 50000 plants) to screen desired mutants. Few successful examples demonstrated the advantage of a large size population in literature. A total of 83083 M_2_ plants were screened for isolating herbicide-resistant (against sulfonylurea herbicide) mutants in lentils (Rizwan et al., 2017). Recently, McMurray et al. (2019) selected two mutant lines (M043 and M009) from 9,500,000 M_2_ population developed from ‘PBA Flash’ variety through ethyl methyl sulphonate (EMS) based mutation breeding. Interestingly, both the mutant lines were tolerant to metribuzin herbicide (a broad-spectrum herbicide affecting photosystem II). Therefore, it is quite evident that induction and effective screening of desired mutants requires an adequate size of a mutagenized population.

### 7.4 Screening methodology for identification of mutants

Mutations are recessive in nature and hence are not visible in M_1_ generation, therefore the screening for mutants with improved agronomical traits including disease resistance in the M_2_ generation is recommended (Mondal et al., 2011; Raina et al., 2017). Single plant harvest of all M_1_ plants may be grown in single row by following the plant to row method with standard spacing. Based on availability of facilities, the M_2_ population may be artificially infested by the pathogens of the targeted disease (Ali and Shaikh, 2007; Ali et al., 2010; Rizwan et al., 2017; Ayala-Doñas et al., 2022). Thereafter, the disease resistant plants may be selected based on the visual performance of the plants in the field. In the earlier generations, breeders often select only high yielding plants with good agronomic features and mutants are artificially screened for targeted disease in advanced generations. Plants selected in M_2_ generation may be grown to raise M_3_ generation followed by screening for a targeted disease to evaluate their true to type behaviour and resistance to disease or targeted traits (Punia et al., 2014; McMurray et al., 2019). While growing the M_3_ population, best susceptible check variety must be grown after every 10^th^ row to create a natural epiphytotic environment (Nene et al., 1981). Based on the availability of pathogens, individual plants may be artificially treated with a critical load of inoculums of the targeted disease (Bravo, 1983; Solanki and Phogat, 2005). Mutants showing resistance to the disease with good agronomic features may be selected for further advancement. If the isolated mutants showed consistent and stable performance in the M_4_ generation, the seeds may be bulked and stored to raise the M_5_ generation and evaluated in replicated yield trials (Laskar and Khan, 2017; Rizwan et al., 2017). Based on their performance, they may be evaluated in multi-location and national trials in M_7_ and M_8_ generations by following appropriate experimental design along with recommended agronomic practices. Multi-location testing may be repeated for 2-3 years to confirm the adaptability and stability of the mutant lines. Based on the performance of mutants in multi-location trials and national trials, the mutant genotype may be recommended for release to a particular location by the state variety release committee or for the whole country by the central variety release committee (Toker et al., 2007). Upon release of the mutant by the technical committee, they may be submitted for notification from the government authority for entering into a quality seed production channel.

### 7.5 Role of mutation breeding for induction of disease resistance in lentil

#### 7.5.1 Possibility for loss of function mutation to behave as disease resistant/tolerant

Pathogen exploit disease susceptibility gene products to gain access into the plant cell and take over replication machinery (Eckardt, 2002). Mutations in these susceptibility genes may disrupt their functions and thus impede the pathogen entry and multiplication inside plant cells and eventually affects pathogenesis. Such types of resistance behave as recessive genes and impart durable broad-spectrum resistance to crop plants (Bravo, 1983; Solanki and Sharma, 2001; Liu et al., 2021; Koseoglou et al., 2022). These resistances are well documented against the virus (mutant *eIF4E* gene in pepper against potato virus Y; Ruffel et al., 2002), fungus (*mlo* in barley for resistance against *Blumeria graminis* f. sp. *hordei*; Brown, 2015), bacteria (*xa13/OsSWEET11* in case of rice for *Xanthomonas oryae* pv. *oryzae* race 6; Yang et al., 2006).

#### 7.5.2 Possibility of gain of function mutations to behave as disease resistant/tolerant

Most of the resistance (R) genes are either non-functional or may play a role in association with other R genes in providing disease resistance. The binding of the pathogen’s avirulence (AVR) gene product on the leucine-rich repeat (LRR) domain of R-protein induces a conformational change that helps binding of ATP in nucleotide-binding site (NBS) domain. Hydrolysis of ATP induces another conformational change in the protein that led to aggregation of R-protein to form either resistosome complex (Wang et al., 2019) or three-dimensional conformation changes in Toll Interleukin-1 Receptor (TIR) domain which hydrolyze NAD+/NADP+ (Horsefield et al., 2019). All these above protein-protein interactions mediate through domain-specific non-covalent interactions between specific amino acids. Thus, changes in any amino acids through point mutations involving non-synonymous mutations in the interacting helix/loops may lead to gain of function.

### 7.6 Present thrust and requirement in mutation breeding for disease resistance

#### 7.6.1 Strategy against stemphyllium blight disease

Stemphyllium blight, caused by *Stemphyllium botryosum* is an important fungal disease that is predominant in all major lentil growing regions. In a recent coordinated effort, FAO-IAEA joint division has formulated a project to induce resistance against Stemphyllium blight in lentil through induced mutagenesis. Cao et al., 2019 recently undertook a leaf transcriptome analysis to detect the differentially expressed genes (DEGs) in resistant and susceptible bulk of a recombinant inbred line population derived from wild lentil species, *Lens ervoides*. This analysis reported several DEGs in resistant plants and an upregulated transcript in susceptible plant/bulk. It was hypothesized that this upregulated gene (codes for uncharacterized protein Lc07593) in susceptible genotypes is a candidate for ‘genes for susceptibility’ in lentil. Mutations can be created in this gene through random mutagenesis/Targeting Induced Local Lesions IN Genomes (TILLING) or targeted mutagenesis approach like clustered regularly interspaced short palindromic repeats and CRISPR associated protein 9 (CRISPR-Cas9) in these genes. These mutants can be bio-assayed in the field or controlled laboratory conditions to detect resistance against *Stemphyllium botrysum*. The same approach could also be followed to induce resistance against rust and Ascochyta blight and anthracnose disease in lentil.

#### 7.6.2 Strategy against pea seed-borne mosaic virus

PSbMV is more common viral diseases and infestation at earlier stage causes a substantial reduction in the seed yield (up to 72%) (Aftab et al., 1992). Viral disease including PSbMV are often transmitted in the field by means of aphids. Eukaryotic translation initiation factor 4E (eIF4E) is exploited by the PSbMV virus to translate its RNA into other viral proteins for multiplication and cell to cell movement. Gao et al. (2004) while working in pea reported that the *sbm1* mediated resistance to two pathotypes P1 and P4 of PSbMV is a consequence of mutations in an *eIF4E* homolog. In contrast, Kang et al. (2005) showed that transient expression of susceptible-*eIF4E* in a resistant background complemented PSbMV infection. The above genetic basis for resistance against PsbMV will pave the way to find mutations or allelic variation in a homologue of *eIF4E* of lentil toward a generation of field resistance through conventional mutation breeding and TILLING approach.

#### 7.6.3. Strategy against fusarium wilt disease (is there any genome editing target)?

Host oxylipin pathways are important for pathogenesis, successful colonization, reproductive development, and biosynthesis of mycotoxins by certain fungal pathogens including *Fusarium* sp. *Fusarium* exploits the jasmonate pathway in plants to create an initial infection. The enzyme lipoxygenase (lox) catalyzes the conversion of α-linolenic acid to its 13-hydroperoxide derivative leading to jasmonate production (Wasternack and Strnad, 2018). Gao et al. (2007) showed that disruption of maize 9-lipoxygenase (*lox 9*) resulted in increased resistance to *Fusarium verticillioides* and reduced levels of fumonisin (a mycotoxin) production. Direct evidence of *in vitro* mutagenesis using ethyl methane sulphonate for wilt resistance also exists in the development of five Fusarium wilt-resistant lines of banana (*Musa* spp., AAA) (Chen et al., 2013). Subsequently, Ghag et al. (2014) identified a down-regulated lipooxygenase (LOX) gene responsible for providing resistance against *Fusarium oxysporum* f. sp. *cubense* in a somaclonal mutant of banana. Lanubile et al. (2021) confirmed the strategic role of *ZmLOX4* in controlling defense against *F. verticillioide* through induction of Mutator-insertion mutagenesis. The above example in the disruption of an isoform of *lox* genes reiterates the practice of mutation breeding for induction of mutations in such equivalent genes in lentil to enhance the resistance against Fusarium wilt without compromising plant vigour and seed yield.

## 8 Role of new breeding technologies in disease resistance breeding

New breeding technologies including genomics assisted breeding (GAB), speed breeding and gene editing, and next-generation breeding targets developing climate-resilient varieties using all sorts of strongly associated marker identification, phenotyping based on machine learning and artificial intelligence (Razzaq et al., 2021). Genomic data along with added information from pan genomes, modification in CRISPR technology, innovation in genome editing and advanced form of base editing were considered for food security in this era of new breeding technologies (Fasoula et al., 2020).

### 8.1 Genomics assisted breeding in lentil

Significant progress in gene-based SSR and SNP markers, availability of draft genome sequence of lentil and cost-effective sequencing of functional regions of lentil genome has made the journey smooth for efficient MAB by virtue of the development of tightly linked markers for disease resistance (Sari, 2014; Sudheesh et al., 2016; Bhadauria et al., 2017a; Bhadauria et al., 2017b; Polanco et al., 2019; Bawa, 2020; Ma et al., 2020). A breeder-friendly marker should have tight linkage having a distance of <1.0 cM from the genes/QTL controlling a trait of interest and explain high phenotypic variation (Collard et al., 2005). Later employment of next-generation sequencing techniques like ‘genotyping by sequencing (GBS)’ has helped in the identification of three nested QTLs on linkage group 5 (9.5-11.5% PVE) and a QTL on linakge group 2 (9.6% PVE) for Ascochyta blight resistance and identification of putative causal genes (Dadu et al., 2021). Ma et al. (2020) used GBS strategy to genotype a RIL population and identified 19 QTLs for Aphanomyces root rot resistance in lentil. In parallel, genome wide association studies (GWAS) were also practiced in lentil for identification of marker-trait association for Aphanomyces root rot resistance (Ma et al., 2020), agronomic traits (Rajendran et al., 2021), prebiotic carbohydrates (Johnson et al., 2021) and salt tolerance (Dissanayake et al., 2021). All these above examples of detecting QTLs/associated SNPs for a targeted trait in lentil have shown promise to apply genomic selection to select genotypes with multiple disease resistance. The concept of GAB evolved to deal with complex traits like yield through involvement of genome-wide markers for selection. The well-characterized training population help to identify such markers for GAB and then applied in a test population after validating them in a subset of training population. In context to disease resistance breeding, GAB will be more helpful to pyramid all the resistance genes in selected plants. Towards this, the Multiparental Advanced Generation Intercross (MAGIC) population involving hybridization among different sources of resistance and elite lines will be helpful to get a genotype with multiple disease resistance through GAB. Whatever products will generate through these above new breeding teachniques (GAB, MAB and GWAS) should be stabilized before testing in a multilocation yield trial. Speed breeding can help to stabilize the selected plants rapidly in a breeding scheme. Normal greenhouse can produce 2-3 generations, whereas rapid generation cycle in speed breeding facilities 4-6 generations in several crops such as wheat, barley, durum wheat, pea and canola (Watson et al., 2018). Manipulating light sources with a very low red:far red ratio was standardized to cause the earliest flowering in lentils (Mobini et al., 2014; 2016). The materials developed in such a speed breeding facility can also be screened for multiple disease resistance and shared with partners for varietal evaluation.

### 8.2 Possible application of gene editing technology for disease resistance breeding

The era of gene/genome editing offers targeted alternations of a particular gene or portion of genome without no alternations in other parts of the genome. Thus the derived product will have same agronomic potential except the targeted change. It offers to rectify some drawbacks of a megavariety within a short span of time. Targeted knockout of negative regulators of disease resistance gene and/or susceptibility genes *via* genome editing tools is a rapid and powerful approach for disease resistance plant breeding (Ahmad et al., 2020). But, before implementing such new techniques in genotype improvement, scientists must take care about possible off-targets through the careful design of guide-RNA. Xu et al. (2019) had demonstrated the successful induction of broad-spectrum bacterial blight resistance by using CRISPR/Cas9 mediated gene editing of two *OsSWEET* genes (S genes) in rice. Further, targeted mutation of *Oryza sativa* ethylene responsive factor 922 (a negative regulator of disease resistance gene) yielded enhanced disease resistance against rice blast (Wang et al., 2016). Such an example in model crop plants shows promise of using gene editing technologies to induce disease resistance in lentils. A working gegetic transformation protocol is a prerequisite to demonstrating these gene editing tools in lentil. Several genetic transformation methods including *Agrobacterium*-mediated genetic transformation have been attempted in lentil (Gulati et al., 2002; Sarker et al., 2012). There are few reports on successful lentil transformation, but transformation efficiency is less than 1.0% (Atkins and Smith, 1997; Sarker et al., 2019). *In vitro* plant regeneration of explants from different lentil tissues, including shoot apices, epicotyls, nodal segments, embryo axes, cotyledonary nodes, and roots, has been attempted for genetic transformation (Mahmoudian et al., 2002; Sarker et al., 2003; Akcay et al., 2009). Cotyledon-attached decapitated embryos appeared to provide the best response toward *in vitro* regeneration following genetic transformation.

### 8.3 Putative candidate disease resistance genes in lentil

WRKY genes are important in disease resistance due to their involvement in several secondary metabolite production and senescence pathways (Yoda et al., 2002). Among several putative candidate genes for disease resistance β-1,3-glucanase, a Bet v I (a pathogenesis-related protein 10), disease resistance response protein homologue of pea, disease resistance response protein G49-C, pathogenesis related protein-4 and antimicrobial protein SNAKIN-2 are fully sequenced lentil disease resistance genes (Kumar et al., 2015). NBS family resistance gene analogue have also been identified in *Lens* species (Yaish et al., 2004). Expression study of defense responsive genes, including pathogenesis-related protein, chitinase etc., have explained their role in plant immunity and can be utilized in genomics lead breeding (Tarafdar et al., 2018). Genomics breeding in lentil was started using orthologous gene information and taking help from a synteny crop like *Medicago tranculata* and *Lotus japonicas* (Weller et al., 2012). EST search-based effector identification revealed CtNUDIX and CtToxB effector are involved in *Collectotrichum lentis* infection (Bhadauria et al., 2013a). In a transcriptome study using wild *Lens ervoides* for Stemphylium blight resistance, various genes of oxidation-reduction process, asparagine metabolism were differentially expressed. Of which, a specifically calcium transporting ATPase and glutamate receptor 3.2 showed differential expression between resistant and susceptible bulk (Cao et al., 2019). CC-NBS-LRR R gene has been identified in the lentil, showing differential expression upon *Colletotrichum lentis* infection (Bhadauria et al., 2013b). Transcriptomic analysis of host-pathogen interaction revealed complex molecular interplay between 26 resistance genes in lentil and 22 effector genes in *Colletotrichum lentis*. Both positive and negative regulators of plant immunity such as suppressor of npr1-1 constitutive 1 (SNC 1) and dirigent as well as markers of antagonistic defense signaling pathways such as PR 1, PR 5 (for salisylic acid mediated pathway) and PR 4 (for jasmonic acid mediated pathway) were found upregulated during the compatible lentil - *Colletotrichum lentis* interaction (Bhadauria et al., 2017b). The challenge remains in identifying the susceptibility genes from these above disease resistance genes in lentil. The future breeding strategy will involve exploiting such S genes in site-directed mutagenesis through gene editing technology.

## 9 Conclusion and future perspectives

Intensive selection pressure for certain agronomic traits on segregating populations derived from hybridization between closely related and common breeding lines has narrowed down the genetic variability of lentil. Crop vulnerability due to the limited genetic variability heightened the risk for biotic and abiotic stresses. Such infestations are turning into disastrous looks due to climatic changes in some pockets of the World. Genetic diversity plays a decisive role in the development of novel plant varieties. Genetic improvement of lentil requires introducing new alleles that extend beyond the existing adapted germplasm pool. New genes and alleles must be identified or generated either through introgression from wild relatives or through induced mutagenesis in lentil genetic resources to attain further breakthroughs in biotic stress resistance with high stability. Induced mutagenesis and site-directed mutagenesis offers a solution for creating new variations and genes. Deployment of CRISPR-Cas9 technology will hasten the process of creating new alleles. Such new breeding technology demands the design of sequence-specific sgRNA cassettes. Availability of reference genome of lentil (Redberry) (https://knowpulse.usask.ca/lentilgenome) will offer a strong foundation for designing such specific sgRNAs towards trait improvement. Gene editing can provide an easier, cheaper, and more precise way of disrupting genes for lentil improvement. Before implementing the new breeding technology for lentil improvement, generating trait variation through induced mutagenesis is essential. Induced mutagenesis offers to understand the nature of mutations and apply the knowledge to rapidly improve the trait through targeted genome editing using CRISPR/Cas9 technology in lentil.

**Figure 4:**
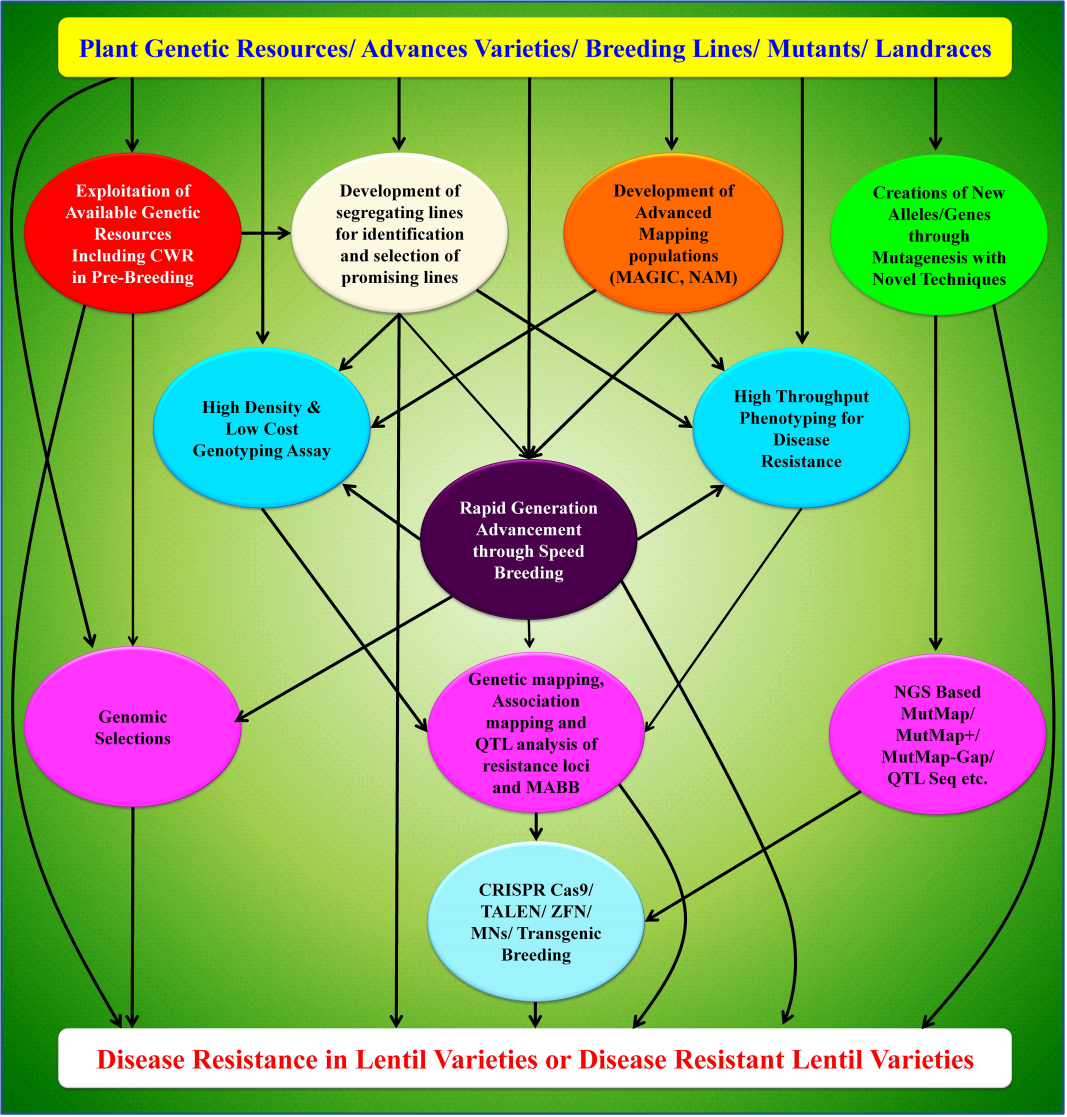
A schematic depicting the future road map for disease resistance breeding in lentil.

Further, deploying precise gene-editing technology in lentil requires good regeneration and efficient transformation protocols. Optimization of the protocol with an appropriate combination of mineral media and hormones is required in near future. Whatever means are there to improve the plant traits, the selections must be stabilized from early generation to near cent percent homozygosity. Rapid generation advancement through speed breeding technique offers a solution to stabilize the generated mutants in lentil in a short span of time. It is possible to stabilize the lentil plant to complete homozygosity within two years through the use of speed breeding technique. Still, there is a scope to improvise this speed breeding protocol in terms of various optimized parameters like type of light-emitting diodes, quality of light, spectral composition and red/far-red light ratio. Resistance breeding in lentil has sufficiently shown a path to exploit the crop wild relatives (CWRs) to better this crop. A schematic depicting the future road map for disease resistance breeding in lentil is presented here (**Figure 4**). Future works must continue in this direction to untap available genetic resources along with CWRs. Such usage can be accelerated by deploying a high throughput phenotyping facility for disease screening in pre-breeding materials. Utilization of elite lines in recombination breeding with pre-breeding materials can be made to generate mapping populations, including MAGIC and Nested Association Mapping (NAM). This will offer to tag the resistance genes and develop more dense flanking markers for disease resistance QTL. Rapid advancement in high-density and low-cost genotyping assay will further help to accelerate the process of marker development and offers great promise in precise genomic selection and/or marker-assisted selection. Integrating advanced mutagenesis tools and speed breeding techniques will further identify new genes/alleles for disease resistance and rapidly develop the varieties. All these new research initiatives lead to developing disease resistance genotypes/varieties that can be deployed to the farmers’ field through productive linkages between research institutes and private institutes/enterprises towards quality seed production.

## Data availability statement

The original contributions presented in the study are included in the article/supplementary material. Further inquiries can be directed to the corresponding authors.

## Author contributions

Abstract: SM. Background/Introduction (Importance of lentil, Recent trends in production, Concepts of disease resistance in general): AnR, and AaR. Biotic production constraints of lentil: AnR, AaR & SM. Available genetic resources to tackle these biotic constraints: AnR, CD. Screening methodologies available for disease resistance breeding: PS. Resistance breeding of lentil (Different breeding approaches, Examples): AnR & PS. Role of mutation breeding in lentil improvement: SM and PS. Role of new breeding technologies in disease resistance breeding: AnR & SM. Conclusion and future perspectives: SM. All authors contributed to the article and approved the submitted version.
